# Nomograms for predicting the overall and cancer-specific survival in patients with non-urothelial carcinoma of the bladder: A population-based study

**DOI:** 10.17305/bb.2023.9881

**Published:** 2024-06-01

**Authors:** Zhenchi Li, Zhibin Xu, Jiangping Wang, Mei Wang

**Affiliations:** 1 Department of Histology and Embryology, Basic Medical College, China Medical University, Shenyang, Liaoning, China; 2 Department of Urology, The Affiliated Taizhou People’s Hospital of Nanjing Medical University, Taizhou School of Clinical Medicine, Nanjing Medical University, Taizhou, Jiangsu, China; 3 Department of Anesthesiology, The Affiliated Taizhou People’s Hospital of Nanjing Medical University, Taizhou School of Clinical Medicine, Nanjing Medical University, Taizhou, Jiangsu, China

**Keywords:** Non-urothelial, bladder cancer, nomogram, Surveillance, Epidemiology, and End Results (SEER)

## Abstract

Non-urothelial carcinoma of the bladder (NUCB) is a relatively rare condition, with limited comprehensive studies conducted to date. This research aims to establish nomograms for forecasting overall survival (OS) and cancer-specific survival (CSS) in NUCB patients. It utilizes data of 2522 patients from the Surveillance, Epidemiology, and End Results (SEER) database spanning from 2004 to 2015. The effectiveness of these nomograms was assessed using receiver operating characteristic (ROC) curves, calibration curves, and decision curve analysis (DCA). Key independent predictors for OS included age, race, marital status, histological variants, grade, T stage, N stage, M stage, radical cystectomy (RC), and chemotherapy administration. For CSS, the predictors were similar, encompassing age, sex, marital status, histological variants, grade, T stage, N stage, M stage, RC, and chemotherapy. The nomograms showed strong predictive accuracy. In the training cohort, the area under the curve (AUC) values were 0.796 (OS) and 0.799 (CSS) at 1-year, 0.807 (OS) and 0.824 (CSS) at 3-year, and 0.807 (OS) and 0.827 (CSS) at 5-year intervals. In the validation cohort, AUC values were 0.798 (OS) and 0.798 (CSS) at 1-year, 0.810 (OS) and 0.826 (CSS) at 3-year, and 0.811 (OS) and 0.825 (CSS) at 5-year intervals, consistently around 0.8. Calibration curves indicated high congruence between the predicted and actual probabilities of OS and CSS, while DCA demonstrated the models’ substantial clinical utility. Overall, this study successfully developed and validated prognostic nomograms for NUCB, capable of accurately predicting OS and CSS at 1, 3, and 5 years, thereby offering valuable support in clinical decision making and the design of clinical trials.

## Introduction

Bladder cancer ranks as the seventh most prevalent cancer among men and the 17th among women worldwide, making it a significant concern in urology [1]. In 2022, it was projected that of the approximately 81,180 new bladder cancer diagnoses, about 17,100 patients would unfortunately succumb to the disease [2]. While the majority of bladder cancer cases are characterized by pure urothelial carcinoma, a minority of patients are diagnosed with non-urothelial carcinoma. This subgroup includes subtypes like adenocarcinoma, squamous cell carcinoma, sarcoma, and others [3, 4]. Non-urothelial carcinoma of the bladder (NUCB) remains poorly understood regarding pathogenesis and biological features compared to urothelial carcinoma [5–8]. The rarity of NUCB has led to its exclusion or underrepresentation in clinical trials, resulting in a lack of robust evidence for treatment protocols and a dearth of specific diagnostic and therapeutic guidelines. Consequently, clinicians often rely on individual case analyses and expert consensus to develop treatment strategies for this subtype of bladder cancer.

In recent years, significant advancements have been made by the scientific community in developing predictive models for various types of malignancies. Prior studies have formulated a nomogram for bladder cancer [9]. Yet, the intricacies of non-urothelial bladder cancer, characterized by a diversity of histological subtypes, clinical stages, and molecular heterogeneity, present distinct challenges in devising reliable and versatile prognostic models. Concurrently, despite its utility, the American Joint Committee on Cancer (AJCC) staging system exhibits limitations in its solo application for accurate prognostic predictions. For patients with NUCB, it is crucial to consider all potential risk factors in tailoring individualized treatment plans. To our knowledge, there is currently no nomogram specifically for prognosticating NUCB, largely due to limited clinical and pathological data. However, the imperative to improve patient outcomes and quality of life underscores the necessity of addressing these challenges. Thus, our study utilized data from a representative cohort of NUCB patients in the Surveillance, Epidemiology, and End Results (SEER) database. We developed two nomograms to evaluate overall survival (OS) and cancer-specific survival (CSS), aiming to provide a prognostic reference for these patients.

## Materials and methods

### Patient information

This study’s patient data were sourced from the SEER database, a public repository offering cancer statistics to reduce the cancer burden. Data extraction and screening were conducted using SEER*Stat software (version 8.4.1.2). Inclusion criteria included: (1) Diagnosis based on the International Classification of Diseases (ICD) for Oncology-3 (ICD-O-3) Topography Codes C67.0-67.9, (2) Identification of four major non-urothelial histological variants of bladder cancer, as classified by the 2016 World Health Organization (WHO) [10], including squamous cell carcinoma, adenocarcinoma, other epithelial tumors, and neuroendocrine carcinoma, and (3) patient age over 18 years. Exclusion criteria involved: (1) patients with unknown or minimal follow-up time (less than one month) to mitigate bias and assure data validity and (2) patients with insufficient clinical and pathological details. The study ultimately included 2522 patients diagnosed with primary NUCB between 2004 and 2015. The dataset included age, sex, race, marital status, histological variant, grade, T stage, N stage, M stage, RC, chemotherapy, radiation therapy, and survival time.

### Ethical statement

As the SEER database is publicly accessible, no ethical committee review or approval was required for using this data. Since all of the clinical data included in this study came from open sources, no federal, state, or institutional regulations are a concern. In addition, informed permission was waived because the data used in this retrospective analysis came from the publicly available SEER database.

### Statistical analysis

Quantitative data with normal distribution were presented as the mean ± standard deviation (SD). In contrast, data with a skewed distribution were described using the median and interquartile range (IQR). Categorical data were expressed as count and percentage (*n*/%). For quantitative data comparison between groups, a two-sample *t*-test was applied for normally distributed data, while a non-parametric rank sum test (Wilcoxon Mann–Whitney test) was used for skewed data. Group differences were evaluated using the appropriate chi-squared and Fisher’s exact tests.

Patients diagnosed with non-urothelial carcinoma were segregated into training and validation cohorts, maintaining a 2:1 ratio. The study incorporated their clinical, pathological, and surgical data in a univariate Cox regression analysis. Variables showing statistically significant differences (*P* < 0.05) were further analyzed using stepwise multiple Cox regression analysis to determine independent risk factors. Nomograms were subsequently constructed via the “rms” package, assessing patients’ OS and CSS based on these factors. These nomograms were validated using a distinct validation cohort. To ensure the model’s reliability and accuracy, particularly in smaller sample sizes, we employed the bootstrap method for internal validation. This involved creating a calibration curve to properly fit the nomogram. The area under the curve (AUC) was derived through receiver operating characteristic (ROC) analysis. The nomogram’s clinical utility was evaluated using decision curve analysis (DCA). Data processing was performed using R software (version 4.2.1, [https://www.r-project.org//]). All tests were two-sided, and a *P* value of less than 0.05 was considered statistically significant.

**Table 1:** The characteristics of non-urothelial carcinoma of the bladder

	**Overall (*N* ═ 2522)**	**Training group** ** (*N* ═ 1765)**	**Validation group** ** (*N* ═ 757)**	**χ^2^**	***P* value**
*Age, years*				0.055	0.814
<65	1035 (41.0%)	727 (41.2%)	308 (40.7%)		
≥65	1487 (59.0%)	1038 (58.8%)	449 (59.3%)		
*Sex*					
Male	1500 (59.5%)	1054 (59.7%)	446 (58.9%)	0.141	0.708
Female	1022 (40.5%)	711 (40.3%)	311 (41.1%)		
*Race*					
Black	266 (10.5%)	189 (10.7%)	77 (10.2%)	0.207	0.902
White	2123 (84.2%)	1482 (84.0%)	641 (84.7%)		
Other	133 (5.3%)	94 (5.3%)	39 (5.2%)		
*Marital status*				2.823	0.244
Married	1366 (54.2%)	973 (55.1%)	393 (51.9%)		
Single	451 (17.9%)	303 (17.2%)	148 (19.6%)		
Other	705 (28.0%)	489 (27.7%)	216 (28.5%)		
*Histological variants*				3.584	0.310
Squamous	1187 (47.1%)	825 (46.7%)	362 (47.8%)		
Adenocarcinoma	716 (28.4%)	498 (28.2%)	218 (28.8%)		
Neuroendocrine carcinoma	420 (16.7%)	291 (16.5%)	129 (17.0%)		
Other	199 (7.9%)	151 (8.6%)	48 (6.3%)		
*Grade*				0.447	0.930
I	288 (11.4%)	197 (11.2%)	91 (12.0%)		
II	648 (25.7%)	453 (25.7%)	195 (25.8%)		
III	988 (39.2%)	696 (39.4%)	292 (38.6%)		
IV	598 (23.7%)	419 (23.7%)	179 (23.6%)		
*T stage*				1.909	0.592
T1/Ta/Tis	741 (29.4%)	515 (29.2%)	226 (29.9%)		
T2	865 (34.3%)	616 (34.9%)	249 (32.9%)		
T3	509 (20.2%)	359 (20.3%)	150 (19.8%)		
T4	407 (16.1%)	275 (15.6%)	132 (17.4%)		
*N stage*				1.379	0.721
N0	2134 (84.6%)	1487 (84.2%)	647 (85.5%)		
N1	182 (7.2%)	133 (7.5%)	49 (6.5%)		
N2	201 (8.0%)	142 (8.0%)	59 (7.8%)		
N3	5 (0.2%)	3 (0.2%)	2 (0.3%)		
*M stage*				0.113	0.736
M0	2237 (88.7%)	1568 (88.8%)	669 (88.4%)		
M1	285 (11.3%)	197 (11.2%)	88 (11.6%)		
*Radical cystectomy*					
Yes	666 (26.4%)	472 (26.7%)	194 (25.6%)	0.339	0.561
No	1856 (73.6%)	1293 (73.3%)	563 (74.4%)		
*Chemotherapy*				0.279	0.597
Yes	842 (33.4%)	595 (33.7%)	247 (32.6%)		
None/Unknown	1680 (66.6%)	1170 (66.3%)	510 (67.4%)		
*Radiation*				1.504	0.220
Yes	425 (16.9%)	308 (17.5%)	117 (15.5%)		
None/Unknown	2097 (83.1%)	1457 (82.5%)	640 (84.5%)		

**Table 2:** Univariate Cox analysis of prognostic factors for overall survival (training group)

**Variables**	**HR**	**95% CI**	***P* value**
*Age, years*			
<65	Reference		
≥65	1.73	1.57–1.90	<0.001
*Sex*			
Female	Reference		
Male	0.83	0.75–0.91	<0.001
*Race*			
Black	Reference		
White	0.81	0.70–0.93	<0.001
Others	0.61	0.47–0.79	0.003
*Marital status*			
Married	Reference		
Single	1.33	1.17–1.51	<0.001
Other	1.68	1.51–1.86	<0.0001
*Histological variants*			
Squamous	Reference		
Adenocarcinoma	0.83	0.74–0.93	0.001
Neuroendocrine	1.36	1.20–1.54	<0.001
Other	0.91	0.76–1.09	0.296
*Grade*			
I	Reference		
II	1.61	1.34–1.94	<0.001
III	2.51	2.11–2.99	<0.001
IV	2.01	1.67–2.43	<0.001
*T stage*			
T1/Ta/Tis	Reference		
T2	2.22	1.96–2.51	<0.001
T3	1.74	1.51–2.01	<0.001
T4	3.58	3.11–4.14	<0.001
*N stage*			
N0	Reference		
N1	1.81	1.54–2.14	<0.001
N2	2.45	2.10–2.86	<0.001
N3	2.57	1.07–6.20	0.035
*M stage*			
M0	Reference		
M1	3.36	2.94–3.83	<0.001
*Radical cystectomy*			
No	Reference		
Yes	0.88	0.79–0.97	<0.014
*Chemotherapy*			
None/Unknown	Reference		
Yes	1.24	1.13–1.37	<0.001
*Radiation*			
None/Unknown	Reference		
Yes	1.86	1.66–2.09	<0.001

HR: Hazard ratio; CI: Confidence interval.

**Table 3:** Univariate Cox analysis of prognostic factors for cancer-specific survival (training group)

**Variables**	**HR**	**95% CI**	***P* value**
*Age, years*			
<65	Reference		
≥65	1.34	1.20–1.50	<0.001
*Sex*			
Female	Reference		
Male	0.71	0.64–0.79	<0.001
*Race*			
Black	Reference		
White	0.73	0.62–0.86	<0.001
Others	0.57	0.43–0.77	<0.001
*Marital status*			
Married	Reference		
Single	1.43	1.24–1.65	<0.001
Other	1.68	1.49–1.89	<0.001
*Histological variants*			
Squamous	Reference		
Adenocarcinoma	0.90	0.79–1.02	0.098
Neuroendocrine	1.43	1.24–1.64	<0.001
Other	0.79	0.63–0.99	0.044
*Grade*			
I	Reference		
II	1.98	1.55–2.51	<0.001
III	3.16	2.51–3.96	<0.001
IV	2.50	1.97–3.18	<0.001
*T stage*			
T1/Ta/Tis	Reference		
T2	3.20	2.72–3.76	<0.001
T3	2.73	2.28–3.26	<0.001
T4	5.45	4.56–6.51	<0.001
*N stage*			
N0	Reference		
N1	2.05	1.72–2.46	<0.001
N2	2.86	2.43–3.36	<0.001
N3	3.20	1.33–7.71	0.009
*M stage*			
M0	Reference		
M1	3.80	3.30–4.36	<0.001
*Radical cystectomy*			
No	Reference		
Yes	0.94	0.83–1.06	0.313
*Chemotherapy*			
None/Unknown	Reference		
Yes	1.39	1.24–1.55	<0.001
*Radiation*			
None/Unknown	Reference		
Yes	1.86	1.63–2.11	<0.001

HR: Hazard ratio; CI: Confidence interval.

## Results

### Clinical and pathological characteristics

A total of 2522 patients with NUCB were included in this study. The majority of the patients were male (59.5%), ≥65 years (59.0%), and white (84.2%). Out of the total, 1366 patients (54.2%) were married. The most common pathological type observed was squamous cell carcinoma (47.1%). In terms of grade, 39.2% and 23.7% of patients were diagnosed with grade III and IV disease, respectively. Among these patients, 70.6% had muscle-invasive disease, without lymph node metastasis (84.6%) and distant metastasis (88.7%). A total of 26.4% of the patients underwent RC. Additionally, 66.6% of patients did not receive chemotherapy and 83.1% did not receive radiotherapy. The training cohort and validation cohort were included in a ratio of 2:1, and there were no statistical differences in the baseline data between the two groups (Table 1).

The majority of the patients were male (59.5%), aged ≥65 years (59.0%), and of white ethnicity (84.2%). Among them, 54.2% (1366 patients) were married. Squamous cell carcinoma was identified as the predominant pathological type (47.1%). In terms of disease grade, 39.2% of patients were diagnosed with grade III and 23.7% with grade IV. A significant portion, 70.6%, presented with muscle-invasive disease. The prevalence of lymph nodes and distant metastases was 84.6% and 88.7%, respectively. RC was performed on 26.4% of the patients. Chemotherapy was not administered to 66.6% of the patients, and 83.1% did not undergo radiotherapy. The training and validation cohorts were proportioned in a 2:1 ratio, with no significant statistical differences in baseline data between the two groups (Table 1).

**Figure 1.**
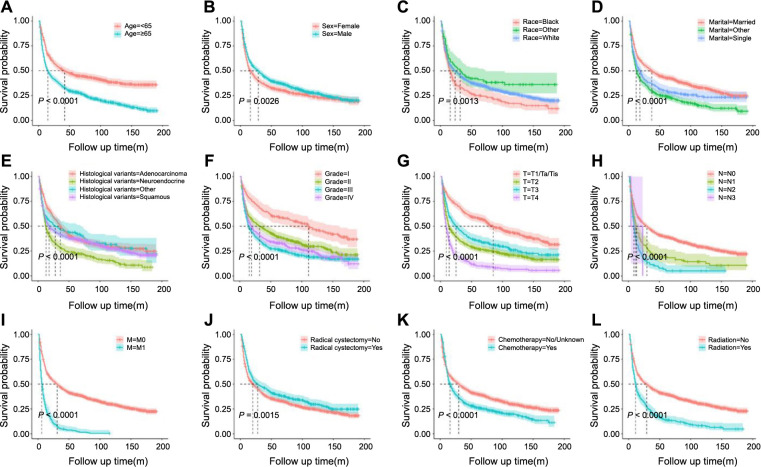
**Kaplan–Meier OS curves of the patients with NUCB according to different clinical characteristics**. (A) Age; (B) Sex; (C) Race; (D) Marital status; (E) Histological variants; (F) Grade; (G) T stage; (H) N stage; (I) M stage; (J) Radical cystectomy; (K) Chemotherapy; (L) Radiation. OS: Overall survival; NUCB: Non-urothelial carcinoma of the bladder.

**Figure 2.**
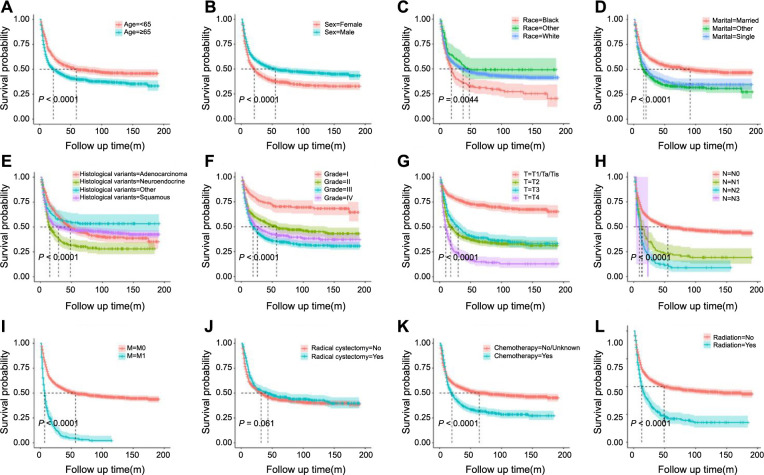
**Kaplan–Meier CSS curves of the patients with NUCB according to different clinical characteristics.** (A) Age; (B) Sex; (C) Race; (D) Marital status; (E) Histological variants; (F) Grade; (G) T stage; (H) N stage; (I) M stage; (J) Radical cystectomy; (K) Chemotherapy; (L) Radiation. CSS: Cancer-specific survival; NUCB: Non-urothelial carcinoma of the bladder.

**Figure 3.**
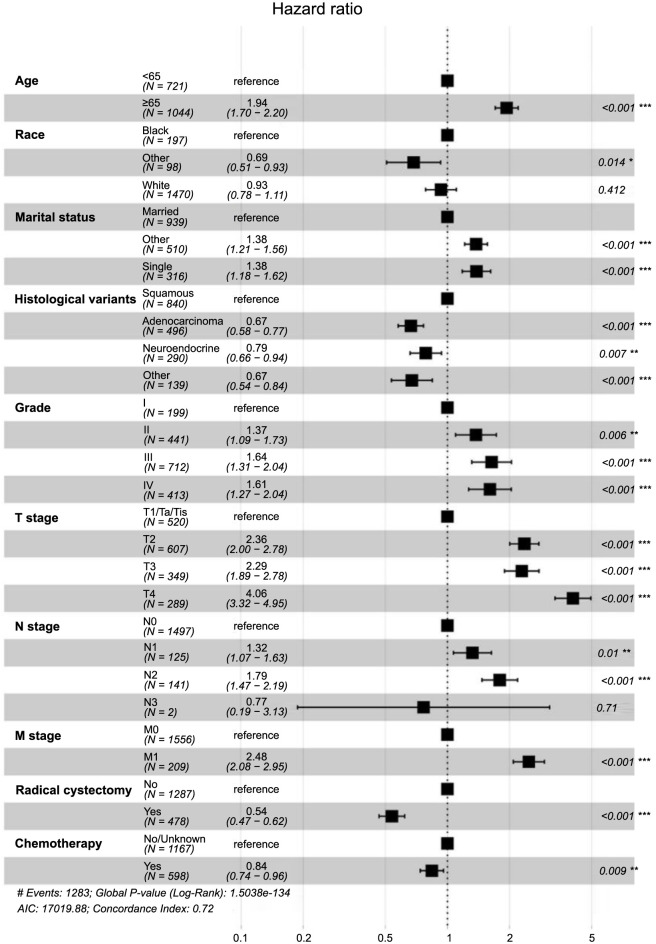
Multivariate Cox analysis of prognostic factors for overall survival (training cohort).

**Figure 4.**
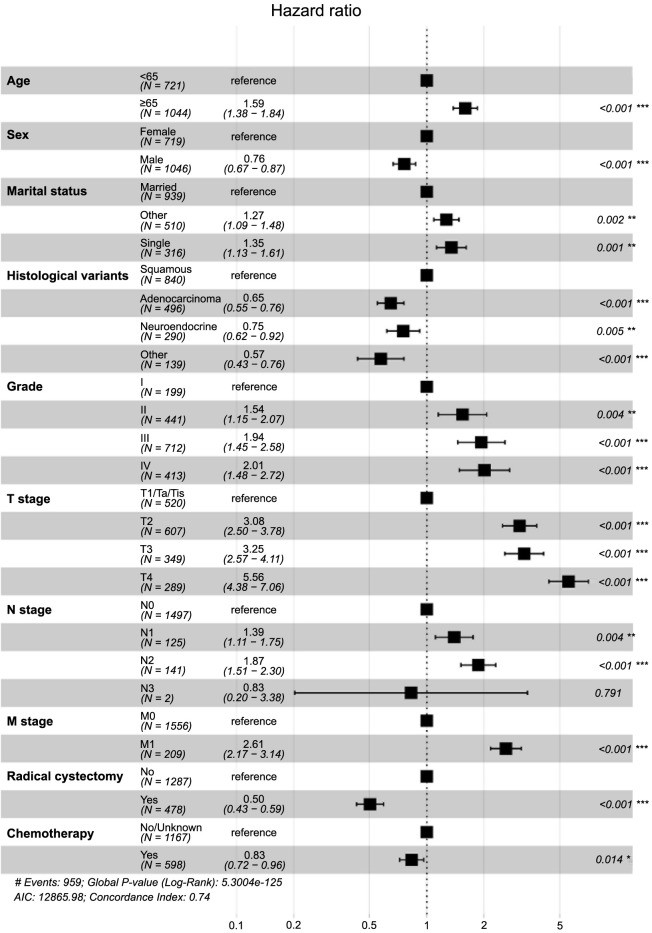
Multivariate Cox analysis of prognostic factors for cancer-specific survival (training cohort).

Univariate and multivariate Cox regression analyses were utilized to identify prognostic factors in the training set. Univariate Cox regression analysis indicated that age, sex, race, marital status, histological variants, grade, T stage, N stage, M stage, radiation, and chemotherapy were significant to patients’ OS and CSS. Although RC’s impact on CSS was not statistically significant (*P* ═ 0.061), it was clinically relevant and, thus, included in the multivariate model (Tables 2 and 3 and Figures 1 and 2). The multivariate regression analysis highlighted age, race, marital status, histological variants, grade, T stage, N stage, M stage, RC, and chemotherapy as independent prognostic factors for OS in NUCB patients (Figure 3). Age, sex, marital status, histological variants, grade, T stage, N stage, M stage, RC, and chemotherapy were identified as independent prognostic factors for intentional survival in NUCB (Figure 4).

The prognostic influencing factors of patients in the training set were determined using univariate and multivariate Cox regression analysis. The results of the univariate Cox regression analysis showed that age, sex, race, marital status, histological variants, grade, T stage, N stage, M stage, radiation, and chemotherapy were all associated with the OS and specific survival prognosis of patients. RC had an impact on OS, but its effect on specific survival was not statistically significant (*P* ═ 0.061) (Tables 2 and 3 and Figures 1 and 2). Despite the oblate significance of RC (*P* ═ 0.061), it was clinically relevant for predicting CSS and was therefore included in the multivariate regression model. The multivariate regression analysis identified age, race, marital status, histological variants, grade, T stage, N stage, M stage, RC, and chemotherapy as independent prognostic factors for OS in NUCB (Figure 3). Additionally, age, sex, marital status, histological variants, grade, T stage, N stage, M stage, RC, and chemotherapy were identified as independent prognostic factors for intentional survival in NUCB (Figure 4).

### Nomogram development

**Figure 5.**
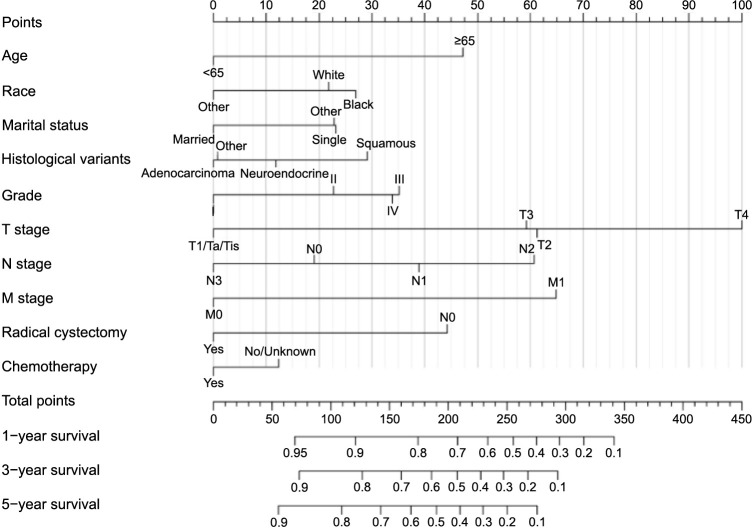
A prognostic nomogram for predicting the overall survival of non-urothelial carcinoma of the bladder patients for the 1, 3, and 5 years.

**Figure 6.**
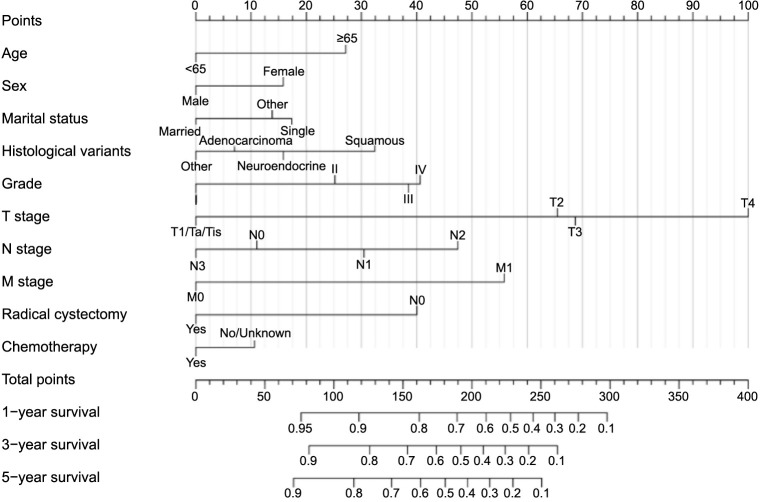
A prognostic nomogram for predicting the cancer-specific survival of non-urothelial carcinoma of the bladder patients for the 1, 3, and 5 years.

This study’s initiative was to construct two comprehensive nomograms by integrating a range of factors: age, race, marital status, histological variants, grade, T stage, N stage, M stage, RC, and chemotherapy. These factors are visually represented in Figures 5 and 6, where each variable’s regression coefficient was depicted by a horizontal line with a corresponding scale, culminating in the construction of columnar lines for both OS and CSS nomograms.

### Evaluation and calibration of nomograms

**Figure 7.**
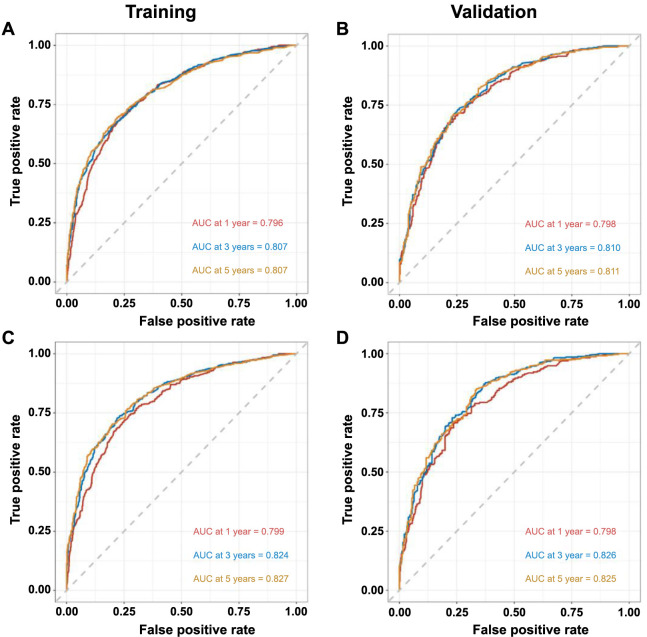
**Time-dependent ROC curves for predicting 1-year, 3-year, and 5-year overall survival in (A) the training cohort and (B) the validation cohort.** Time-dependent ROC curves for predicting 1-year, 3-year, and 5-year cancer-specific survival in (C) the training cohort and (D) the validation cohort. ROC: Receiver operating characteristic; AUC: Area under the curve.

**Figure 8.**
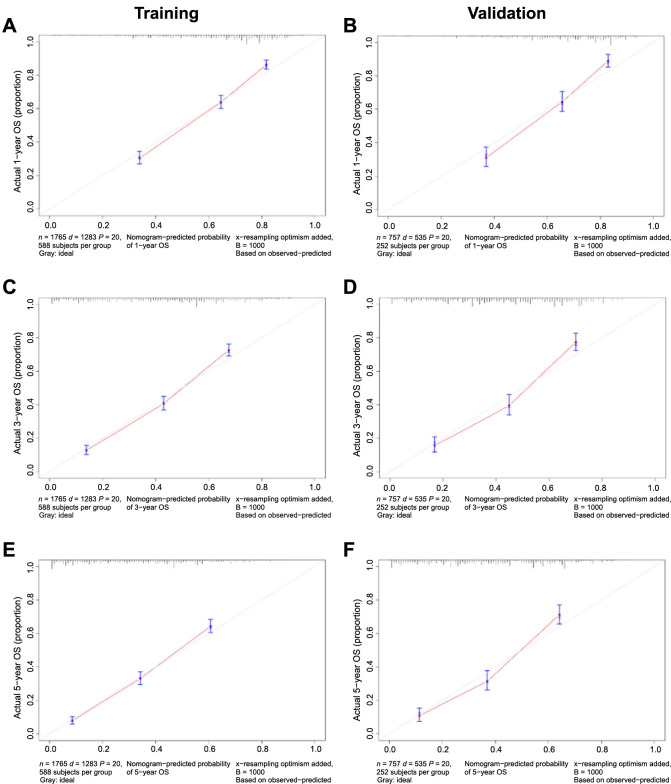
**Calibration curves of internal validation for 1-, 3-, and 5-year overall survival (A, C, E) in the training cohort and (B, D, F) in the validation cohort.** OS: Overall survival.

**Figure 9.**
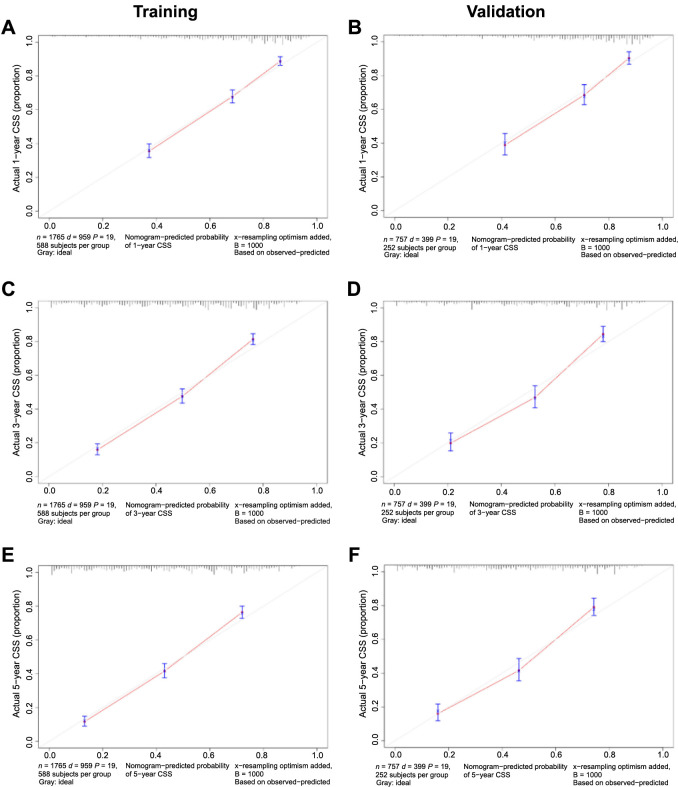
**Calibration curves of internal validation for 1-, 3-, and 5-year cancer-specific survival (A, C, E) in the training cohort and (B, D, F) in the validation cohort.** CSS: Cancer-specific survival.

Discrimination of these nomograms was evaluated using ROC curves. In the training cohort, the AUC values for 1-year, 3-year, and 5-year OS were 0.796, 0.807, and 0.807, respectively (Figure 7A). In the validation cohort, the AUC values for 1-year, 3-year, and 5-year OS were 0.798, 0.810, and 0.811, respectively (Figure 7B). Regarding CSS, the training set displayed AUCs of 0.799, 0.824, and 0.827 for 1-year, 3-year, and 5-year intervals, respectively (Figure 7C), while the validation set exhibited AUCs of 0.798, 0.826, and 0.825, respectively (Figure 7D). Calibration curves, which were plotted to determine model accuracy, revealed a high concordance between predicted and actual survival outcomes across 1-year, 3-year, and 5-year intervals, indicating robust predictive validity (Figures 8 and 9).

### Clinical decision evaluation

**Figure 10.**
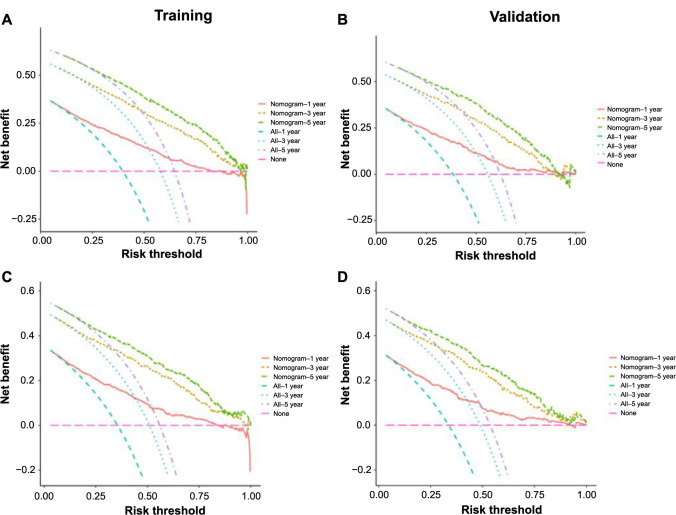
**The DCA of the nomogram for 1-year, 3-year, and 5-year** **overall survival (A) in the training cohort and (B) in the validation cohort; Cancer-specific survival (C) in the training cohort and (D) in the validation cohort.** DCA: Decision curve analysis.

Figure 10 shows a visual analysis of intervention outcomes, comparing scenarios where all patients received an intervention against those where no patients did. Within the 0–1 probability threshold, the model demonstrated a higher net benefit compared to scenarios of either intervention or universal intervention for all patients. This finding suggests that all patients could potentially benefit from this model, emphasizing its clinical applicability.

## Discussion

NUCB accounts for a mere 10%–25% of bladder cancer cases and is characterized by a high degree of invasiveness [11]. Its origin is often attributed to the differentiation of non-urothelial stem cells or the progression of urothelial cell metaplasia [12]. The European Association of Urology recognizes non-urothelial histologic variants as the highest-risk subgroup, often correlating with poorer oncologic outcomes. Retrospective studies consistently indicate that these variants are associated with poor prognoses [13–15]. Given their rarity and challenging prognosis, it is imperative to develop tailored treatment strategies for NUCB patients. Currently, due to the absence of specific guidelines for NUCB, treatment approaches are often adapted from those used for pure urothelial bladder cancer, complicating the development of personalized treatment strategies. While the AJCC staging guidelines have traditionally been the standard for cancer patient assessment and treatment guidance [16], they do not account for the impact of various other factors on patient prognosis. Consequently, accurately assessing the prognosis of NUCB is essential for enhancing patient quality of life. Leveraging data from the SEER database, this study identified key prognostic influences and risk factors, culminating in the development of the first OS and CSS nomograms for NUCB. These tools are invaluable for personalized risk assessment and management, offering significant assistance in clinical decision making, especially in scenarios with limited clinical data.

Sex, age, and race are recognized as independent risk factors for the incidence and progression of bladder cancer [17, 18]. In our study, male patients exhibited a 1.5-fold higher prevalence than female patients, yet they presented with a more favorable prognosis, corroborating findings from existing research. Globally, men are three to five times more susceptible to bladder cancer than women [19]. However, women tend to develop more aggressive tumors, often progressing to muscle invasion, resulting in mortality rates nearly double those of men. This disparity suggests that bladder cancer is often diagnosed at a later stage in women [20]. Initial symptoms of bladder cancer in women are frequently mistaken for infections, leading to delays in proper diagnosis and treatment. Consequently, this results in a poorer prognosis for female patients compared to male patients [21]. Additionally, sex hormones, particularly estrogen and its receptors, including estrogen receptor 1 (ER1), estrogen receptor 2 (ER2), and G-protein-coupled estrogen receptor-30 (GPR30), have been implicated in the pathogenesis and progression of bladder cancer in females, as detailed in prior studies [22]. These studies have also demonstrated that age is a significant factor in the recurrence or progression of bladder cancer and is considered an independent prognostic factor [23]. In our analysis, the age was categorized at 65 years, a threshold reflecting the prevalence of government-provided medical insurance in the United States. Notably, 59% of our study cohort were aged 65 or older, with survival rates decreasing with advancing age. Racial distribution among participants was predominantly white (84.2%), with black patients comprising 10.5%. This group exhibited poorer OS and CSS compared to white patients, a disparity potentially stemming from genetic, lifestyle, occupational, and environmental differences [24]. Additionally, married patients generally show improved survival rates [25], likely due to enhanced social support affecting treatment adherence and mental health. Consistent with these findings, our research observed a poorer prognosis for single individuals compared to married ones. Marital status may act as a surrogate for the intricate interaction of social support, though further research is necessary to unravel these associations. Disparities in healthcare access and preventive measures for advanced disease among black patients may also contribute to poorer outcomes [26].

Non-urothelial carcinomas generally have a poorer prognosis than pure urothelial carcinomas [27]. RC is advised for bladder cancer patients with muscle invasion. In the case of non-muscle invasive bladder cancer, RC is recommended unless contraindicated or declined by the patient. Our findings indicate that RC can improve both OS and CSS in patients. Research by Kim et al. [28] revealed a higher incidence of T3-4 tumors and lymph node metastases in non-urothelial carcinomas compared to urothelial carcinomas (70% vs 38%, *P* < 0.0001 and 20% vs 15%, *P* < 0.05, respectively). Similarly, a study by Takemoto et al. [29] found that patients with non-urothelial carcinoma, predominantly squamous cell carcinoma, were more likely to present with advanced tumor stages and vascular invasion compared to those with pure urothelial carcinoma (*P* < 0.01).

Given the aggressive nature and adverse prognosis of non-urothelial carcinoma, it is advisable to conduct thorough consultations with patients diagnosed with this condition and consider early RC. Additionally, radiation therapy may serve as an alternative for patients in advanced clinical stages or for those unable to undergo surgery [30]. In our study, the majority of patients did not receive radiation (83.1%), but those who did (16.9%) demonstrated improved overall and CSS outcomes. This observation may be attributed to a significant proportion (57.4%) of patients with stage III–IV disease who received radiotherapy. These patients often have poor physical conditions or advanced disease, limiting their suitability for surgery and leading to comparatively less favorable treatment responses. In recent years, nomograms have emerged as prominent prognostic tools in medical research and clinical practice. Their principal entails constructing a multi-factor regression model where each variable is assigned a score reflecting its impact on the outcome. A score is determined for each level of the influencing factor and the total score is calculated by summing the individual scores. The functional conversion relationship between the total score and the probability of the outcome event is used to calculate the predicted probability of the individual outcome. This model is graphically represented as a nomogram. Previous studies have developed nomograms for bladder cancer OS and CSS using data from the SEER database [9, 31], encompassing all bladder cancer types, including both urothelial and non-urothelial tumors. However, this inclusive approach may introduce a potential bias in prognosis estimation. To our knowledge, our study is the first to propose a prognostic nomogram specifically for non-urothelial carcinoma, a rare bladder carcinoma subtype. We identified ten risk factors for OS and CSS through multivariate regression analysis: age, race, marital status, histological variants, grade, T stage, N stage, M stage, RC, and chemotherapy. Two nomograms were constructed for OS and CSS, demonstrating strong predictive performance. In both models, the AUC values for 1-year, 3-year, and 5-year intervals in the training group were 0.796, 0.807, and 0.807 and 0.799, 0.824, and 0.827, respectively, with validation group AUCs also around 0.8, indicating excellent predictive power. The calibration curves affirmed the consistency between the actual and predicted probabilities for overall and specific survival in NUCB. The DCA further highlighted the clinical benefits of these nomograms. This predictive model facilitates vigilant patient monitoring and timely interventions to mitigate poor prognoses.

However, our study is not without limitations. The SEER database lacks certain proven risk factors, such as smoking, occupational carcinogen exposure [32], and environmental exposure [33], which could have enriched our analysis with deeper insights. Furthermore, due to the rarity of non-urothelial carcinoma, there is a paucity of external data for further validation of our findings, underscoring the need for additional external validation to reinforce our conclusions. Thirdly, it is imperative to acknowledge that our primary data source, the SEER database, lacks specific molecular or genetic information. The significance of these aspects is recognized, and we advocate for additional research in this area, ideally utilizing datasets that encompass molecular and genetic data. Furthermore, it is crucial to note that our study is retrospective. Further research involving a larger sample size and prospective study design is necessary to draw more definitive conclusions. Despite these limitations, our research offers significant insights into the prognosis and individualized treatment of patients with NUCB. We believe that our findings make a meaningful contribution to the field, even within the context of these constraints.

## Conclusion

Employing data from the SEER database, this study represents the first retrospective analysis to identify risk factors for OS and CSS in patients with NUCB. Additionally, we have developed and validated nomograms for both OS and CSS, which have demonstrated high accuracy as survival prediction tools and possess considerable clinical application value. These nomograms can be invaluable aids for medical professionals, assisting in interpretation and application in clinical practice.

## Data Availability

The datasets generated and analyzed during the current study are available in the Surveillance, Epidemiology, and End Results (SEER) database (http://seer.cancer.gov/data/sample-dua.html).
